# Supplementary material for: Magnesium modulates phospholipid metabolism to promote bacterial phenotypic resistance to antibiotics
Source: eLife. 2025 Jan 2;13:RP100427. doi: 10.7554/eLife.100427 (PMC11695056; doi:10.7554/eLife.100427)
Supplement: Figure 3—source data 1. [file elife-100427-fig3-data1.zip › Figure 3-source data 1/Figure 3-source data 1.pdf]

FabA

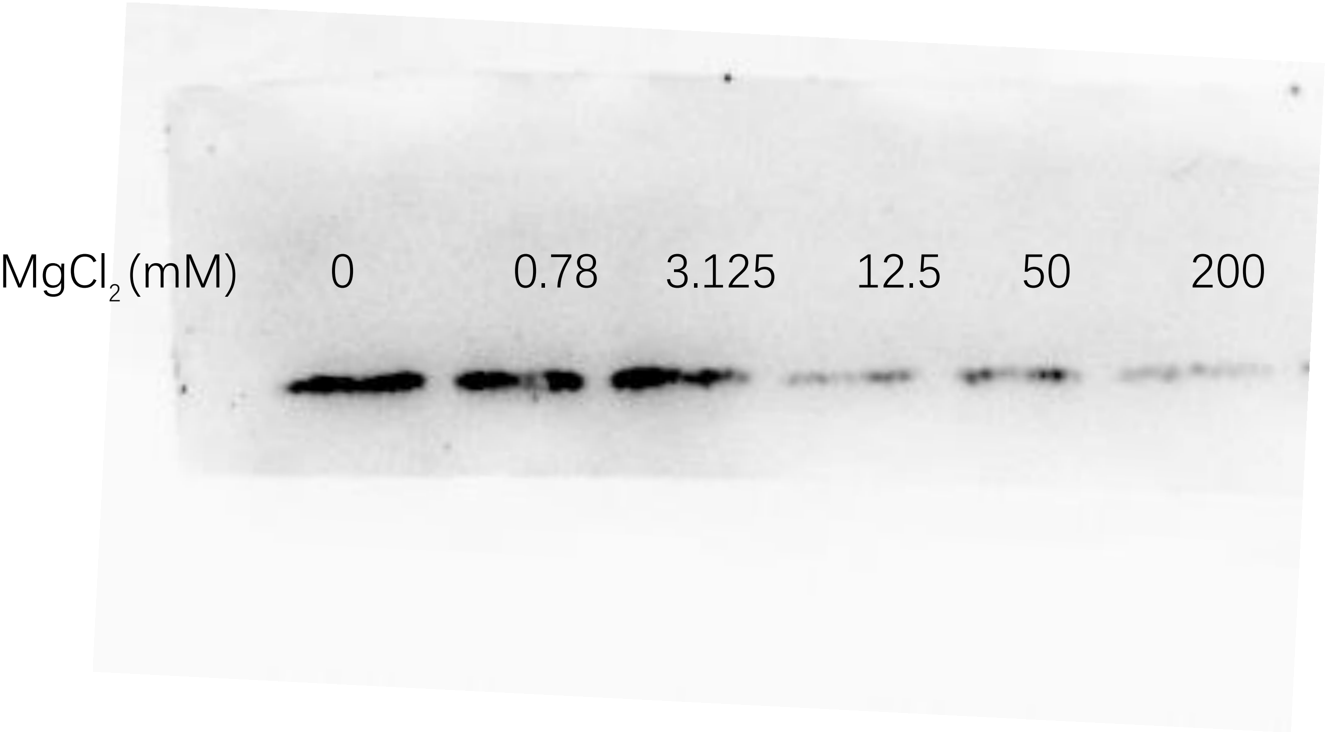

FabF

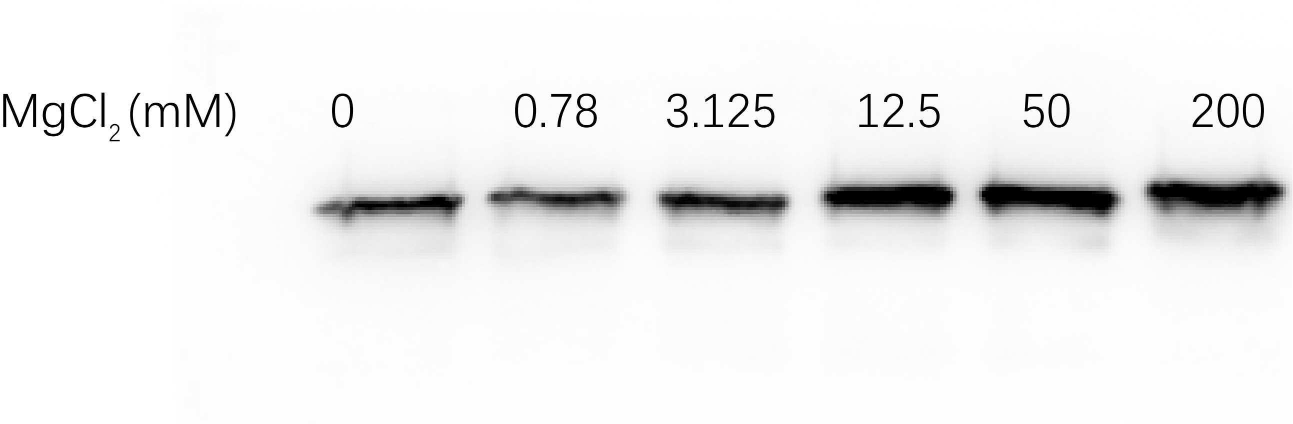

FadL

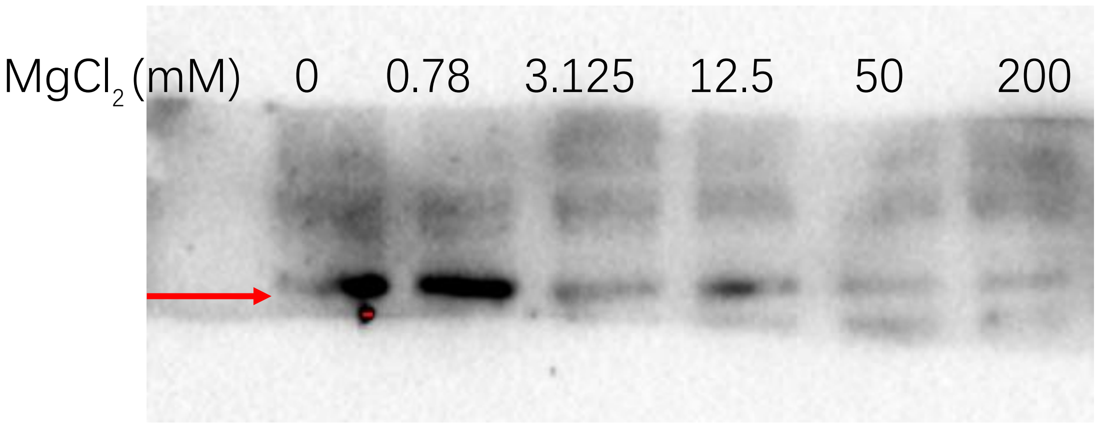

FadR

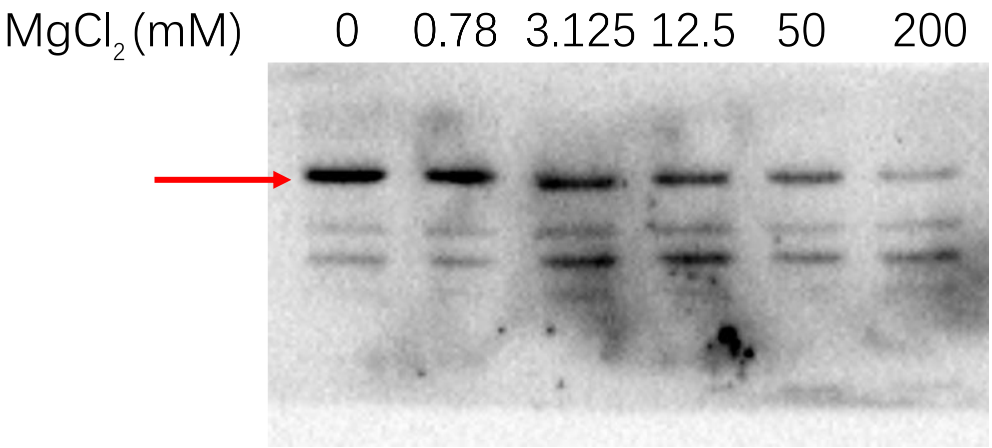

Figure 3. source data 1. Original membranes corresponding to Figure 3D (FabA and FabF), Figure 3I (FadL) and Figure 3K (FadR). SDS-PAGE gels were cut with one part used to stain with Coomassie blue as loading control, while the other part were used for western-blot. Red arrows indicate the location of protein of interest.
