# Supplementary figures and images for: Magnesium modulates phospholipid metabolism to promote bacterial phenotypic resistance to antibiotics
Source: eLife. 2025 Jan 2;13:RP100427. doi: 10.7554/eLife.100427 (PMC11695056; doi:10.7554/eLife.100427)

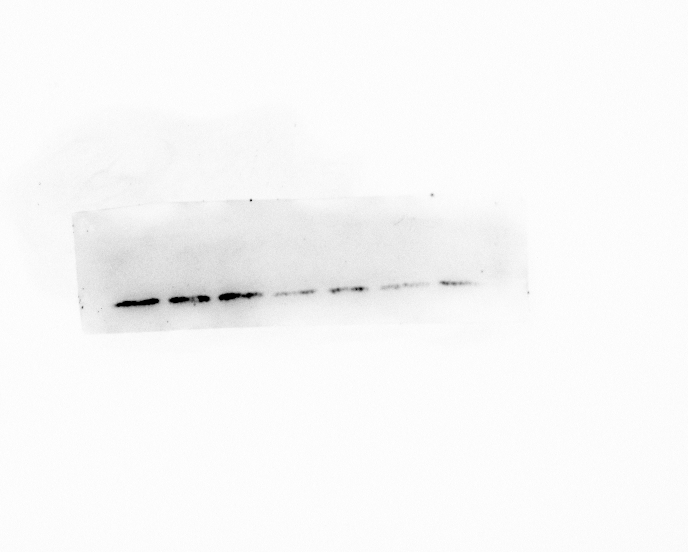

Supplement: Figure 3—source data 2. [file elife-100427-fig3-data2.zip › Figure 3-source data 2/FabA.tif]

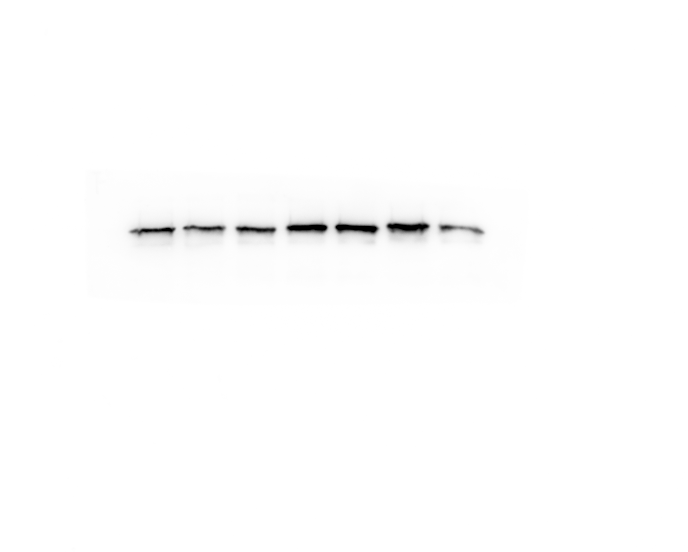

Supplement: Figure 3—source data 2. [file elife-100427-fig3-data2.zip › Figure 3-source data 2/FabF.tif]

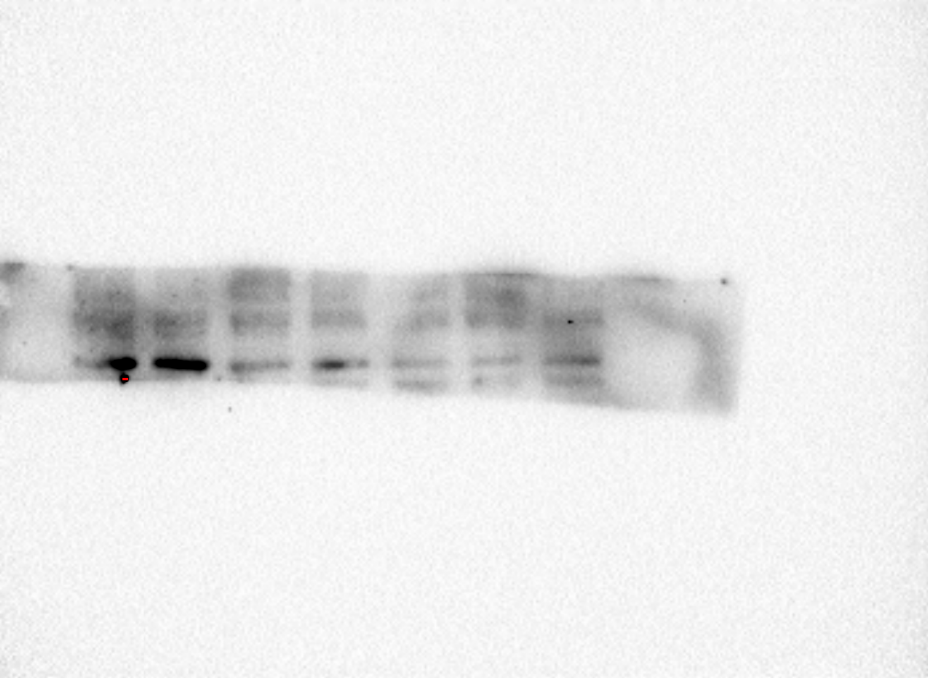

Supplement: Figure 3—source data 2. [file elife-100427-fig3-data2.zip › Figure 3-source data 2/FadL.tif]

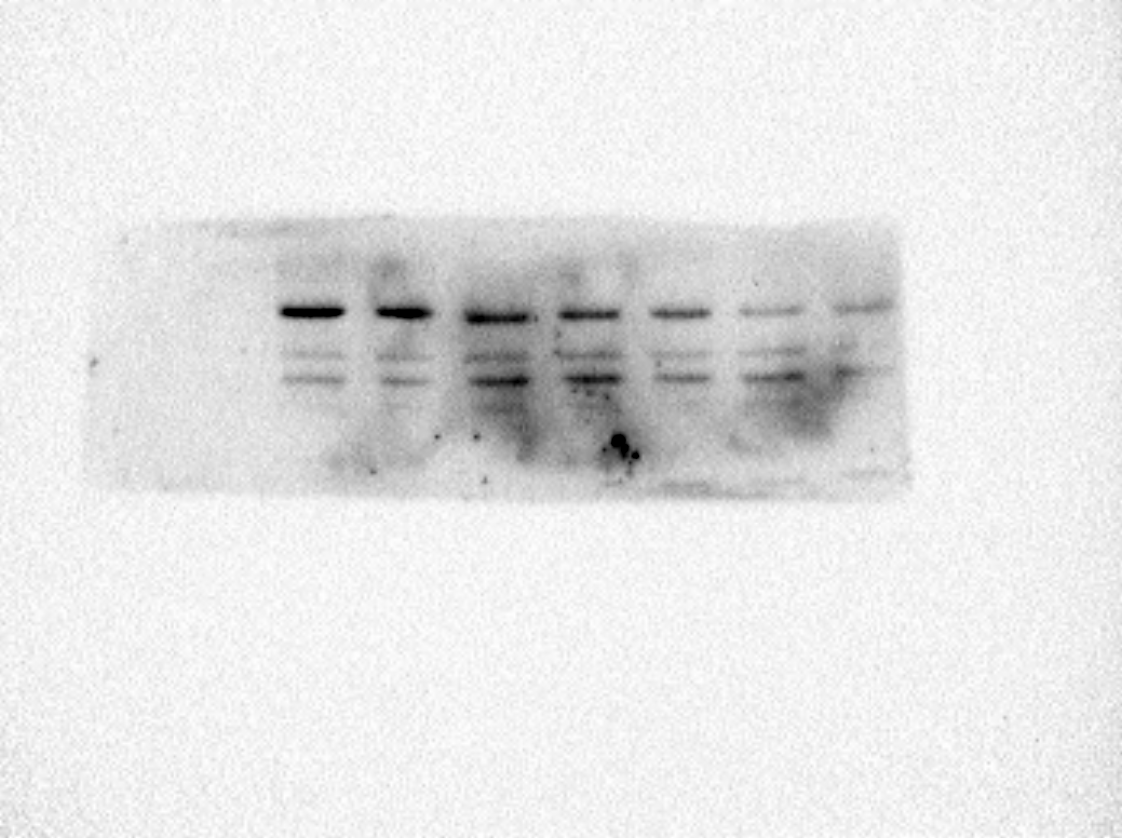

Supplement: Figure 3—source data 2. [file elife-100427-fig3-data2.zip › Figure 3-source data 2/FadR.tif]

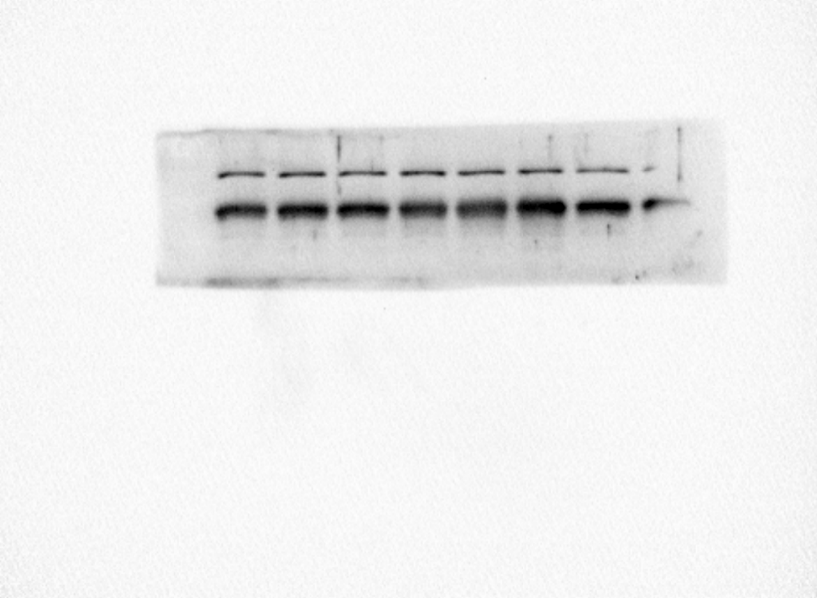

Supplement: Source data 2. [file elife-100427-data2.zip › Appendix 1-source data 2/TolC.tif]
