# Supplementary material for: Magnesium modulates phospholipid metabolism to promote bacterial phenotypic resistance to antibiotics
Source: eLife. 2025 Jan 2;13:RP100427. doi: 10.7554/eLife.100427 (PMC11695056; doi:10.7554/eLife.100427)
Supplement: Source data 1. [file elife-100427-data1.zip › Appendix 1-source data 1/Appendix 1-source data 1.pdf]

TolC

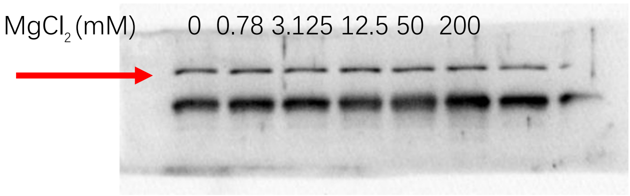

Appendix 1. source data 1. Original membranes corresponding to Figure 1D (TolC). SDS-PAGE gels were cut with one part used to stain with Coomassie blue as loading control, while the other part were used for western-blot. Red arrows indicate the location of protein of interest.
