# Supplementary material for: Magnesium modulates phospholipid metabolism to promote bacterial phenotypic resistance to antibiotics
Source: eLife. 2025 Jan 2;13:RP100427. doi: 10.7554/eLife.100427 (PMC11695056; doi:10.7554/eLife.100427)
Supplement: Supplementary file 1. — (a) Comparison in components in LBS and ASWT. (b) Primes used in the present study. (c) Primers used in the present study for the construction of gene-deleted mutants. [file elife-100427-supp1.docx]

**Supplementary File 1a. Comparison in components in LBS and ASWT**

|  | **LBS** | **ASWT** | |
| --- | --- | --- | --- |
| Tryptone(%) | 1 | | 1 |
| Yeast extract(%) | 0.5 | | 0.5 |
| NaCl(mM) | 171 | | 210 |
| MgSO_4_(mM) | 0 | | 35 |
| KCl(mM) | 0 | | 7 |
| CaCl_2_(mM) | 0 | | 7 |

**Supplementary File 1b. Primes used in the present study**

| gene |  | KEGG entry | Sequences (5’-3’) |  | gene |  | KEGG entry | Sequences (5’-3’) |
| --- | --- | --- | --- | --- | --- | --- | --- | --- |
| *accA* | Forward | N646_1411 | AAAGGTCGTGAAACCAAAGA |  | *fadD* | Forward | N646_4025 | CATTATTTACTGCGATCCTCG |
|  | Reverse |  | AGCCGTGTCGATAAATGTG |  |  | Reverse |  | CCATATTGAGATATTTGTCTGGG |
| *accB* | Forward | N646_1967 | TCAAGCACCAATTCACTACG |  | *fadE* | Forward | N646_1395 | TATCCCAACTGATGTCAAAGG |
|  | Reverse |  | ACTTTCTGACCCACTTCTACG | |  | Reverse |  | CAACCGACAGACACTCAACC |
| *accC* | Forward | N646_1968 | AGAAGTGACTGGTGCTGTGG |  | *fadB* | Forward | N646_2210 | ATCACTAAAACGCCCAGAAAA |
|  | Reverse |  | GGATAGTGTCTGCCTTTGGAC | |  | Reverse |  | GCTGAAGCCGCCAAAGTA |
| *accD* | Forward | N646_1292 | CGAACTTGAGCCACAGGATA |  | *fadA* | Forward | N646_1311 | CAGATTAGCGGCCAGTTTCT |
|  | Reverse |  | ACGACTGAACCCATTGAACC |  |  | Reverse |  | TCAAGCGGCGGTTAATGT |
| *accD* | Forward | N646_4704 | AAGTTTGTCCGAGCAGTTGA |  | *fadA* | Forward | N646_2209 | ATTGCGGGCTGCGATC |
|  | Reverse |  | GAGACGCCACCATAAGTTTG |  |  | Reverse |  | GCACACGGCAGAGACTGC |
| *fabD* | Forward | N646_1131 | CTCGCCTGGTCAAGTCGT |  | *fadH* | Forward | N646_1258 | AAGTGCTGCCCTGCTCG |
|  | Reverse |  | TGCTGGCTTCATAAGTGCA |  |  | Reverse |  | ATGATGGCGTCGAGTTAATG |
| *fabH* | Forward | N646_1132 | GACCGTTCAACCATCATCCT |  | *fabR* | Forward | N646_2038 | TTTGCTACTACGCCAACTGATGCGT |
|  | Reverse |  | GCCATTTATCCGCATCTTG |  |  | Reverse |  | CGCTCACGCAACAACAAACGAA |
| *fabH* | Forward | N646_1439 | GACGCTCCAACATGGCTAG |  | *fadR* | Forward | N646_1147 | AATGCTAGGCGCTGGAA |
|  | Reverse |  | AGTTCGCTGGTGACAATAAGA | |  | Reverse |  | TGAATGCGGCTTCCTG |
| *fabH* | Forward | N646_4350 | GGAAGACAACGGTTACTGGG |  | *arcA* | Forward | N646_1744 | TGGGTTGAATGGTTTAGTGATG |
|  | Reverse |  | TGCGGAATCACAACATCG |  |  | Reverse |  | TGAAGCCAGTGACGGTGAA |
| *fabB* | Forward | N646_1292 | TTGACAGTGCGCCCATC |  | *crp* | Forward | N646_1004 | AAGGTTGCTGCTACTTGCTGA |
|  | Reverse |  | TGTCTTGCTACGCCATTTAAGA | |  | Reverse |  | GCTTCGGATTGGTAATGGTT |
| *fabB* | Forward | N646_3036 | CAACATTAATGCTGCGGCT |  | *crp* | Forward | N646_1885 | CAAAACAACCAGACGCAATG |
|  | Reverse |  | TGAGCGGCCCACATTAC |  |  | Reverse |  | CTCACGAGAACAGCCAACAAT |
| *fabF* | Forward | N646_1128 | GGTGTTGCTATCGGCTCA |  | *gpsA* | Forward | N646_1922 | GTGCGGGTATGTCTGATGG |
|  | Reverse |  | ATGTTTACAATGGTCGAAGGA |  |  | Reverse |  | ATAAAGGTTTCTGGCTGTGC |
| *fabF* | Forward | N646_3033 | AACTGCCAAAGCACTACAAGC | | *plsB* | Forward | N646_2043 | GCTTAGTTCGCTTTAGTCCC |
|  | Reverse |  | TGATGAGCCGTATGCGATAC |  |  | Reverse |  | CACTTGGCGTTGTGGC |
| *fabG* | Forward | N646_1130 | CAACCTAACGCCAATCTACC |  | *plsY* | Forward | N646_2566 | CGCCAAGAATGATAGGGTC |
|  | Reverse |  | CCACGAGACGCAACTTCA |  |  | Reverse |  | TTATTAGGTTCGGTTTCCAGT |
| *fabG* | Forward | N646_3034 | GACCGAAGAAGAATGGGATG |  | *plsC* | Forward | N646_1749 | CGATTCGCTCGGTCAA |
|  | Reverse |  | TGCGCTGTAGTTAGTTTGACC | |  | Reverse |  | TACTGTTTCTGCGGCTGT |
| *fabG* | Forward | N646_3826 | AGTAAAGGCGGCGTCATC |  | *cdsA* | Forward | N646_1423 | TTGCTGCCCTTGTGCC |
|  | Reverse |  | TCTGGCAGGTTCTTGGTCA |  |  | Reverse |  | GCCAAGATTTCGTCGTTTT |
| *fabG* | Forward | N646_4505 | CCCAAGGCGTTATCATCA |  | *pgsA* | Forward | N646_1026 | GTTTTGGCGCGTTTATT |
|  | Reverse |  | TTCGGGTTTCATTTGCTCT |  |  | Reverse |  | TTTCTCGCGCAATCATC |
| *fabZ* | Forward | N646_1416 | GAGAAGTACCTTCACGCGAT |  | *pgpA* | Forward | N646_2838 | ATGAGTTTGCGGGCTTT |
|  | Reverse |  | CTGTTGCCTGAGCCATTG |  |  | Reverse |  | TCCCGCCACGATATCAT |
| *fabV* | Forward | N646_4267 | AGTAATGGGCGGCGAAG |  | *pgpB* | Forward | N646_3562 | TTTCTTTGGCGGGTTGT |
|  | Reverse |  | TGGGTGCGTAATGTCAGG |  |  | Reverse |  | CTTCAGGGCGGTGCAT |
| *fabV* | Forward | N646_0267 | GGTGGTTTACTCACTGGCATC | | *pssA* | Forward | N646_2008 | GATAAAACCGCCAACGA |
|  | Reverse |  | TCTTCACCGCCCATTACC |  |  | Reverse |  | TTACGTGCGGCGATATT |
| *tesA* | Forward | N646_4268 | AGCATTTCTGGCGATACAAC |  | *psd* | Forward | N646_1916 | CACTCTGCGCCAAATG |
|  | Reverse |  | GGAGGGAAACCTCTTAGGC |  |  | Reverse |  | ACGAGGCGGAGTGATC |
| *tesB* | Forward | N646_0021 | CTTTGGATTCGTGCTAATGG |  | *pldA* | Forward | N646_4183 | CTTTTTAGGTCGCCAGAAC |
|  | Reverse |  | ATAGAGTGGTCAATGGTGGC |  |  | Reverse |  | CATAACCCGCTGAGTATTGT |
| *yciA* | Forward | N646_1052 | TGTCTCAGCTCGACTTAGCG |  | *pldB* | Forward | N646_2069 | GATCTCTACCGCCAAGGT |
|  | Reverse |  | GGGTTTGACCCATACTTCCA |  |  | Reverse |  | AGTGCGCGATGATGAAG |
| *fadL* | Forward | N646_1315 | CTAAGTATTGGCGCAACGTACG | | *glpQ* | Forward | N646_1500 | CTATCCAACCCGATTCCC |
|  | Reverse |  | GCCGCCGACTTGTTCTG |  |  | Reverse |  | ACCAAGGCGCTTTAATTT |
| *fadL* | Forward | N646_1316 | CTGCCTTCGCCGATGAT |  | *glpQ* | Forward | N646_3581 | AAAAGTCCAATGCCGCA |
|  | Reverse |  | GAAATTCGCGATTGGTTTG |  |  | Reverse |  | GTAACCGGCTTGTTGGAG |
| *fadL* | Forward | N646_3657 | TTATCTGCGGGTGCCTCT |  | *glpQ* | Forward | N646_3723 | CACAAATGGCGCTATCTG |
|  | Reverse |  | GTCACGCCCGTCACCAT |  |  | Reverse |  | CCATCGGCTTCTTCACTC |
| *fadD* | Forward | N646_1045 | TGTTCTTGAGCTGGTGGAAA |  | *glpQ* | Forward | N646_3724 | GACTTGTGAAGCCCGC |
|  | Reverse |  | AACACGCAATGACGAGGTAT |  |  | Reverse |  | AGATACTCAGGGCCAATAAAC |
| *fadD* | Forward | N646_2540 | GCATCGGTCGATTCTTCC |  | *etuC* | Forward | N646_3725 | CTTTTAAGCGCTGATGATGA |
|  | Reverse |  | CGCCAATCTTAACCTGTGC |  |  | Reverse |  | GTTAGACCGGGTCTCTCTC |
| *fadD* | Forward | N646_3023 | CTCTGATGATGCCAAACCTAC | | *adhE* | Forward | N646_1240 | AGCGATGCTGCACGAA |
|  | Reverse |  | ACGAGGAGTGTAAAGTGGGTT | |  | Reverse |  | CCTGTAACTAATATGGCTGAAC |

**Supplementary File 1c. Primers used in the present study for construction of gene-deleted mutants**

| *gene* |  | primer (5'-3') |
| --- | --- | --- |
| *fadR* | P1 | aaaaaggatcgatcct TACTTCCCCTGAACTCAAGCAC |
|  | P2 | TGTCAGCGCTTATCTACTTTTTATCCAATATTGATTTCTG |
|  | P3 | ATAAAAAGTAGATAAGCGCTGACAAGTTGGATAAAC |
|  | P4 | atcgcatgcggtacc GCAAAGAGATGACTTGCATAATGAGTA |
|  | P5 | ATGGTCATAAAGGCGAAAAGCC |
|  | P6 | TTAGCTGTCGTCTTCCGTGAAGTT |
|  | P7 | AATAGTGCTTAAGTATCTGTTTGT |
|  | P8 | ATGACCATAGTGTTGCAAGA |
| *arcA* | P1 | aaaaaggatcgatcct GACCTCAAGTTACTGTATGGGTTCC |
|  | P2 | TAGCGGTTAAATTGGCGGTACCTAAATTTGTGACA |
|  | P3 | TAGGTACCGCCATAGATTTAACCGCTAATATGCAAAGGG |
|  | P4 | atcgcatgcggtacc ATTGATGGCAGGCTGTAGTTCC |
|  | P5 | ATGCAAACCCCGCAGATTCT |
|  | P6 | TGGCGATGATTTCTGGCGT |
|  | P7 | CGACGGCAAAGCTTTCCTC |
|  | P8 | AAAAGTTGGTTGGAACAAAGGAA |
| *plsB* | P1 | aaaaaggatcgatcct TTAGGTGTTCCTCTGCGGCAT |
|  | P2 | AAGGTAAAACGGAAAGTTCACAGCCTCAATAGGTATTC |
|  | P3 | GCTGTGAACTTTTAACCGTTTTACCTTCTTCCAATAAGA |
|  | P4 | atcgcatgcggtacc AGCTATTTTTAGGCTTGGCTTTTA |
|  | P5 | ACAACGATAATGATGTCCCAACC |
|  | P6 | GAACGACCACCCTCACTGAAGTA |
|  | P7 | GCTCATCATTGGCTGCGTCT |
|  | P8 | TGCTGACCATGAACCCGC |
| *waaf* | P1 | aaaaaggatcgatcct AGTTGTGGGTTCGCTGTGTTGA |
|  | P2 | GAACTAACATGAGCTTTGTTTTTCCAGTATAGGAATT |
|  | P3 | GAAAAACAAAGCTCATGTTAGTTCGTATTGTTTATACCTT |
|  | P4 | atcgcatgcggtacc TTGGCGTAGTTCTGACACGATT |
|  | P5 | AACGCCATCCAGATAGCCAAAT |
|  | P6 | AGGTTTCAAGCCCGCCAAGA |
|  | P7 | ATATGTGGGTACTACAGTTGCTGCA |
|  | P8 | CATCTAATACCTGTTCGTCCTC |
| *lpxA* | P1 | aaaaaggatcgatcct TACGACCTGTTGCCACGTTTG |
|  | P2 | ATTCGCCAATTAATTAAAACTCTCGACGAGCACAC |
|  | P3 | GAGAGTTTTAATTAATTGGCGAATGAACCAATAAATAA |
|  | P4 | atcgcatgcggtacc ATTTTAAAGATGCGCTTCTGACG |
|  | P5 | GGCAGCGGTGGTAGAAGAGG |
|  | P6 | AACGGGTGAATCGCAGACAG |
|  | P7 | ATTGATGAGATGAATAAGCGCCT |
|  | P8 | TAACTCGCTACCACGACTGCCT |
| *lpxC* | P1 | aaaaaggatcgatcct AGACGGTTGGTAATACAGTGAAG |
|  | P2 | GCCAAGTATTTACTGCTTGCCTCTCAAAAATTTGC |
|  | P3 | AGAGGCAAGCAGTAAATACTTGGCATTAGAATTAATAA |
|  | P4 | atcgcatgcggtacc CTGGAGAAATCTTACAGCTTAAC |
|  | P5 | ATTGAATCAGACGAGCAACG |
|  | P6 | CGAATGTAGTCCACTCCCAC |
|  | P7 | AGAATGCCGTTCAAGGTATTGC |
|  | P8 | TTTACTTAAAGTGGTGACTGATT |
| *pgpA* | P1 | aaaaaggatcgatcct AAGCACTAGAGCGAGCTCATTAC |
|  | P2 | GTTCATGGTTTAATCCTTAAACCTATACTGCTAGTTT |
|  | P3 | AGGTTTAAGGATTAAACCATGAACTAAACACTGGACGT |
|  | P4 | atcgcatgcggtacc ATACCCTCGTGGTAGCGGCAT |
|  | P5 | GGCACAATTACCATTTACGG |
|  | P6 | TAGAAACAGGGCAACACCAG |
|  | P7 | CATTTTTCGAACTGGCTGATT |
|  | P8 | GCAACGTGGTTATGACCAATT |

Small letters are homologous recombination sequences with pDS132 plasmid.
